# AFM/TIRF force clamp measurements of neurosecretory vesicle tethers reveal characteristic unfolding steps

**DOI:** 10.1371/journal.pone.0173993

**Published:** 2017-03-21

**Authors:** Mark C. Harris, Dillon Cislo, Joan S. Lenz, Christopher Umbach, Manfred Lindau

**Affiliations:** 1 School of Applied and Engineering Physics, Engineering, Cornell University, Ithaca, NY, United States of America; 2 Department of Materials Science and Engineering, Cornell University, Ithaca, NY, United States of America; 3 Laboratory for Nanoscale Cell Biology, Max-Planck-Institute for Biophysical Chemistry, Göttingen, Germany; UPR 3212 CNRS -Université de Strasbourg, FRANCE

## Abstract

Although several proteins have been implicated in secretory vesicle tethering, the identity and mechanical properties of the components forming the physical vesicle-plasma membrane link remain unknown. Here we present the first experimental measurements of nanomechanical properties of secretory vesicle-plasma membrane tethers using combined AFM force clamp and TIRF microscopy on membrane sheets from PC12 cells expressing the vesicle marker ANF-eGFP. Application of pulling forces generated tether extensions composed of multiple steps with variable length. The frequency of short (<10 nm) tether extension events was markedly higher when a fluorescent vesicle was present at the cantilever tip and increased in the presence of GTPγS, indicating that these events reflect specifically the properties of vesicle-plasma membrane tethers. The magnitude of the short tether extension events is consistent with extension lengths expected from progressive unfolding of individual helices of the exocyst complex, supporting its direct role in forming the physical vesicle-plasma membrane link.

## Introduction

The fusion of secretory vesicles with the plasma membrane occurs from a tethered state, in which the vesicles are associated with the plasma membrane via long-range interactions that do not require the cytoskeleton [1]. This earliest stage of association between the vesicle and the plasma membrane is poorly understood and the nanomechanical properties of these tethers are not known [2]. Vesicle-plasma membrane tethering is followed by docking, priming, and finally vesicle fusion. The precise distinction between tethering and docking has not been clear and consistent in the literature, due in part to limitations of electron microscopic studies [3]. One suggestion is that the vesicle and target membrane are held together within >25 nm in the tethered state, and 5–10 nm in the docked state [4]. Additionally, the docked state is widely thought to involve trans-pairing of Soluble N-ethylmaleimide sensitive factor (NSF) Attachment Protein Receptors (SNAREs) [5, 6], which can only begin to form when the vesicle-plasma membrane distance is below 8 nm [7]. The SNARE proteins in the vesicle and plasma membrane form a coiled coil, and unzipping of the SNARE complex using optical tweezers results in ~8.3 nm extension [8]. Therefore, we consider docking to be a state that involves trans-pairing of SNAREs, while tethering precedes trans-pairing of SNAREs and involves other proteins. The primed state is thought to involve docking with partial SNARE complex assembly making the vesicle readily releasable [3, 9, 10].

Multiple protein complexes have been implicated in exocytosis. Among them are long coiled-coil proteins and multi-subunit tethering complexes (MTCs) [1]. Many MTCs contain CATCHR (Complex Associated with Tethering Containing Helical Rods) domains, characterized by an extended rod-like structure composed of helical bundles [1, 11]. These CATCHR domains promote an elongated structure and mediate interactions with other proteins, such as GTPases. Among the CATCHR proteins that have been implicated in exocytosis are the exocyst complex, Conserved Oligomeric Golgi (COG) complex, Dsl1 complex, Golgi-associated Retrograde Protein (GARP) complex, Calcium-dependent Activator Protein for Secretion (CAPS), Munc13, and the class V myosin Myo2 [1]. The HOPS (Homotypic Fusion and Vacuole Protein Sorting) complex, which likely provides the tethering required for homotypic fusion between large organelles, lacks CATCHR domains. The exocyst complex [12] is a leading candidate as the physical tether between neurosecretory vesicles and the plasma membrane [13]. GTP-binding proteins, such as members of the Rab family, have also been implicated in tethering [14]. Some examples of Rab proteins acting on tethering factors are: Sec4 acting on the exocyst, Ypt7 acting on HOPS, Rab1 acting on p115, and Rab5 acting on EEA1 [3].

Atomic force microscope (AFM) and optical trap pulling experiments have provided much information about protein structure and dynamics. AFM force clamp experiments on reconstituted proteins result in stair-step patterns in which each step corresponds to the unfolding of a single domain, and more mechanically stable proteins take longer to unfold [15]. Optical trap pulling experiments on reconstituted SNARE proteins successfully stabilized a half-zippered state of the SNARE complex and measured the extension change related to zippering of different domains [8]. However, such experiments have been restricted to reconstituted proteins.

Since the molecular identities of the proteins that form the physical tether between neurosecretory vesicles and the plasma membrane are still unknown, we used an AFM to apply a force clamp directly to secretory vesicles tethered to plasma membrane sheets prepared from PC12 cells using a sonic pulse [16, 17]. Membrane sheets prepared in this way retain fusion competent tethered vesicles [16, 17, 18]. The length and frequency of tether unfolding events was measured by the AFM cantilever while total internal reflection fluorescence (TIRF) microscopy was used to locate vesicles and track the movement of vesicles attached to the AFM cantilever tip within the TIRF evanescent wave. The experiments revealed a distribution of tether extension steps around ~5 nm consistent with sequential unfolding of helical domains as found in members of the CATCHR family.

## Results

### Vesicle clusters are found only on membrane sheets

**Fig 1:**
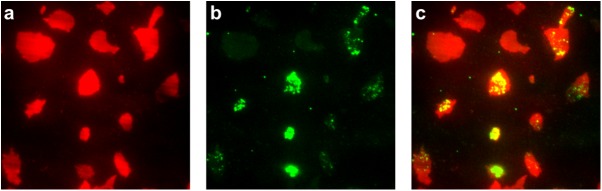
Imaging of membrane sheets. Contrast-enhanced image of ANF-eGFP-tagged secretory vesicles on PC12 membrane sheets labeled with FM 4-64 and excited by 561nm (**a**) and 488nm (**b**) laser light in TIRF mode. Panels (**a**) and (**b**) show the same region of the sample. The 561nm light excites FM 4-64, which labels membrane sheets, while the 488nm light excites eGFP. The overlay (**c**) shows that not all membrane sheets have vesicles on them, but all vesicle clusters are seen on membrane sheets.

Plasma membrane sheets were prepared by application of a sonic pulse to PC12 cells stably expressing eGFP labeled proatrial natriuretic factor (ANF-eGFP) that were cultured on glass coverslips. ANF-eGFP is specifically targeted to peptidergic secretory vesicles in neurons and endocrine cells [19]. To locate membrane sheets for force clamp experiments, membrane sheets were labeled with 0.5 μM FM 4-64. Like other FM dyes, FM 4-64 partitions into membranes and is highly fluorescent there, whereas its fluorescence is quenched in the aqueous phase [20, 21, 22]. FM 4-64 has absorption and emission peaks of 560 nm and 767 nm, respectively, making it suitable for use in cells that express a GFP construct [22, 23]. Although not all membrane sheets had vesicles on them, vesicle clusters were only present on membrane sheets (Fig 1). In the force clamp experiments described below, membrane sheets were thus identified by the presence of vesicle clusters, without need for FM 4-64 staining.

### AFM/TIRF recordings reveal stepwise tether extensions

**Fig 2:**
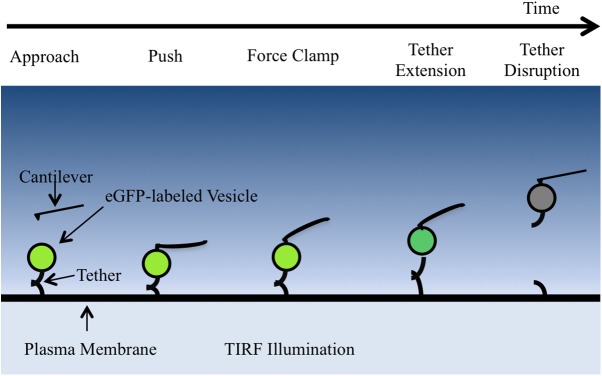
Force clamp procedure. Steps of a force clamp experiment are shown from left to right. First, the cantilever approaches the membrane sheet. After contact, the cantilever tip maintains a push force and binds nonspecifically to the cytoplasmic surface or a vesicle on the surface. The cantilever then applies a pull force (force clamp), which can result in extension or disruption of vesicle-plasma membrane tethers. Increases in vertical position of vesicles within the TIRF evanescent wave result in decreases in fluorescence intensity. Figure not to scale. The cantilever tip radius is approximately half of a vesicle radius.

A method incorporating both Atomic Force Microscopy (AFM) and Total Internal Reflection Fluorescence microscopy (TIRF) was developed to identify the nanomechanical properties of vesicle-plasma membrane tethers by force clamp measurements (Fig 2). The cantilever was lowered onto the membrane sheet having GFP labeled tethered vesicles on its exposed cytoplasmic surface. Following the approach, a push phase was initiated, during which the cantilever could bind nonspecifically to the cytoplasmic membrane surface or a vesicle on the surface. During the subsequent force clamp phase, the cantilever was retracted to apply 4 different pulling forces of increasing magnitude sequentially, each for 12.5 s. If a vesicle moved vertically within the evanescent wave, the extension could also be detected as a change in fluorescence intensity.

**Fig 3:**
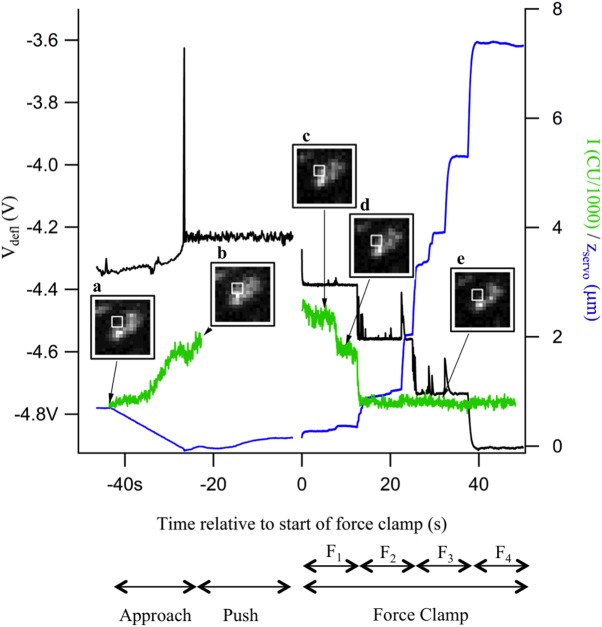
Example of force clamp data acquisition. *V*_*defl*_ (black), *z* (blue), and *I* (green) are plotted on the same time axis, showing the approach, push, and force clamp phases F_1_-F_4_ of the experiment. Insets from the TIRF imaging show the fluorescence changes as a vesicle approaches the surface (**a,b**) with the cantilever tip and is then pulled away (**c-e**). The green trace shows the time course of the fluorescence intensity in the region marked by the white squares in **a-e**.

Forces between the sample surface and the cantilever tip were recorded as deflection of the cantilever, *V*_*defl*_, (Fig 3, black trace), together with the vertical position, *z*, of the cantilever positioning servo (Fig 3 blue trace) and TIRF fluorescence images (insets a-e). For each force clamp experiment, the TIRF recordings were examined for evidence of a fluorescent vesicle at the cantilever tip. The white squares enclose a 3x3 pixel area in which a fluorescence change associated with force application was evident. The average intensity, *I*, of those 9 pixels in every frame of the TIRF image sequence was plotted on the same time axis as the AFM data (Fig 3, green trace).

During the approach phase, the AFM servo was lowered (Fig 3, blue trace), and *I* increased (Fig 3, green trace) as the cantilever approached the surface, because in this experiment, a vesicle was attached to the cantilever tip at the start, presumably picked up in a preceding interaction with the membrane sheet. An increase in *V*_*defl*_ occurred (Fig 3, black trace) as the cantilever made contact, followed by a brief spike when control of the z servo position switched from direct position control to automatic adjustment of position to maintain constant force (constant *V*_*defl*_). The brief spike (if present) was limited to 1 data point and thus shorter than the data acquisition rate during the approach phase (~140 ms per point). Once contact had been established, a constant push force was applied by holding *V*_*defl*_ constant while the inital TIRF image acquisition sequence was stopped and a new acquisition started.

During the force clamp phase of the experiment, 4 different pull forces, F_1_-F_4_, increasing stepwise in magnitude were applied, each for 12.5 seconds, as indicated at the bottom of Fig 3. Tether extension events appeared as transient spikes in the *V*_*defl*_ trace, accompanied by stepwise increases in the *z* trace. When a tether extension event occurred, *V*_*defl*_ increased as the cantilever tip was suddenly released and the cantilever deflected upwards, away from the sample surface. To return *V*_*defl*_ to the setpoint value, the positioning servo moved the cantilever away from the surface, revealing a wide range of tether extension lengths from <10 nm to >1 μm. Along with the first few events in segment 1, an associated decrease in *I* occurred (Fig 3, green trace and insets c-d) due to movement of the vesicle away from the surface into a region of lower intensity of the evanescent wave excitation. After a further intensity decrease at the start of segment 2, no further significant change was seen in *I* because the vesicle had been pulled out of the evanescent wave, leaving only background intensity from surrounding vesicles or ambient light.

### Identification of vesicle-plasma membrane tether extensions

Changes in AFM servo z position may not only reflect vesicle-plasma membrane extensions, but could alternatively be due to detachment of the membrane sheet from the surface or due to extensions of the AFM tip-vesicle link. However, the fluorescence of the vesicles surrounding the vesicle pulled out of the evanescent wave remained unchanged (Fig 3A–3E), confirming that the change in *I* corresponded to a vesicle pulled away from the membrane sheet, and not the membrane sheet itself being detached from the surface.

On the other hand, extension of the AFM tip-vesicle link would not be associated with a change in the vesicle’s TIRF intensity. To quantitatively compare the change in vesicle z position estimated from the change in TIRF intensity with the extension length reported by the AFM servo, the dependence of the vesicle fluorescence intensity *I*_*vesicle*_ on the height z above the surface

$$I_{vesicle}=I_{vesicle,0}\;exp(-z/d_{TIRF})$$ (1)

was used, where *I*_*vesicle*,*0*_ is the value of *I*_*vesicle*_ at z = 0 and *d*_*TIRF*_ is the decay length of the evanescent TIRF excitation wave. The background intensity *I*_*background*_ was determined from the baseline level after the vesicle was pulled out of the evanescent wave, which in the example of Fig 3 occurred after the transition from segment 1 to segment 2. The change in vesicle height Δz from *z*_*i*_ to *z*_*f*_ was thus calculated from the intensities as:

$${\Delta z}_{TIRF}=-d_{TIRF}\times ln\left(\frac{I_{f}-I_{background}}{I_{i}-I_{background}}\right)$$ (2)

**Fig 4:**
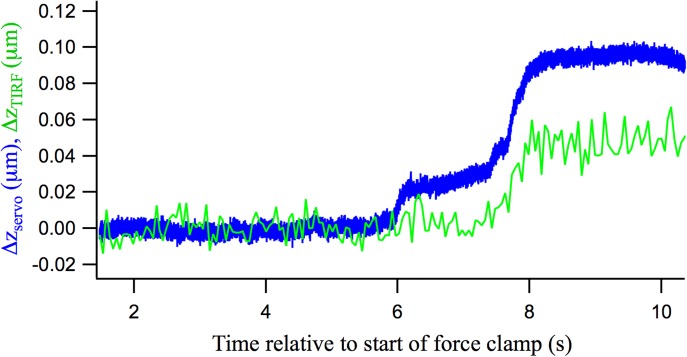
Comparison of AFM servo and fluorescence intensity change. Extension change due to the events in segment 1 of Fig 3, measured by z servo extension (*Δz*_*servo*_), and by conversion of TIRF intensity (*Δz*_*TIRF*_). A strong correlation between the traces is seen in the later phase, but little correlation with the first change in *Δz*_*servo*_.

The servo position change, *Δz*_*servo*_ (Fig 4, blue trace), for the events that occurred between insets c and d of Fig 3 was partially correlated with the extension length calculated from the changes in *I*, *Δz*_*TIRF*_ (Fig 4, green trace).

The late phase of *Δz*_*servo*_ starting at ~7.5 s was matched by a corresponding *Δz*_*TIRF*_ increase of similar magnitude, whereas the initial *Δz*_*servo*_ occurring from ~6–7.5 s was not associated with a change in *Δz*_*TIRF*_. The initial change in the *Δz*_*servo*_ trace did thus not involve movement of the vesicle, but the later extensions did. The initial *Δz*_*servo*_ change therefore appears to be related to an extension of the link between AFM tip and vesicle, whereas the later extensions indicate extensions of the vesicle-plasma membrane tether.

Such a detailed quantitative analysis of the TIRF intensity changes was rarely possible because individual tether extension events were usually too small (< 10 nm) to produce a measurable intensity change. However, a large number of events showed a fluorescent vesicle at the AFM tip that dimmed associated with AFM tip retraction. For analysis, if a fluorescence change was observed in a 3 pixel by 3 pixel region near the cantilever tip over the course a force clamp experiment, that recording was labeled as +ΔFluo, indicating the presence of a fluorescent vesicle. Otherwise, the experiment was labeled as –ΔFluo. In the latter category, a direct AFM tip-plasma membrane interaction may have occurred that did not involve a vesicle, or the vesicle fluorescence may have been too dim to be detected.

### Multiple types of tether extensions

**Fig 5:**
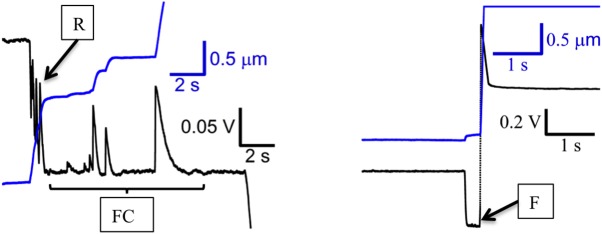
Classification of tether extension event types. Traces show *V*_*defl*_ (black) and *z*_*servo*_ (blue). FC (force clamp) events occur after *V*_*defl*_ setpoint is reached. R (rapid) events occur before the *V*_*defl*_ setpoint is reached. F events occur when the tip dissociates fully from the surface.

For further analysis, the tether extension events were grouped into three categories (Fig 5). Type FC (force clamp) events occurred during the force clamp, after the set-point *V*_*defl*_ for the segment was reached. Type R (rapid) events occurred before the set-point *V*_*defl*_ for the segment was reached. They were characterized by noticeable transients in the deflection trace. Type F (full dissociation) events were characterized by a sudden increase in *V*_*defl*_, after which there was no evidence of a pull force and reflect a full dissociation of the cantilever tip from the surface.

**Fig 6:**
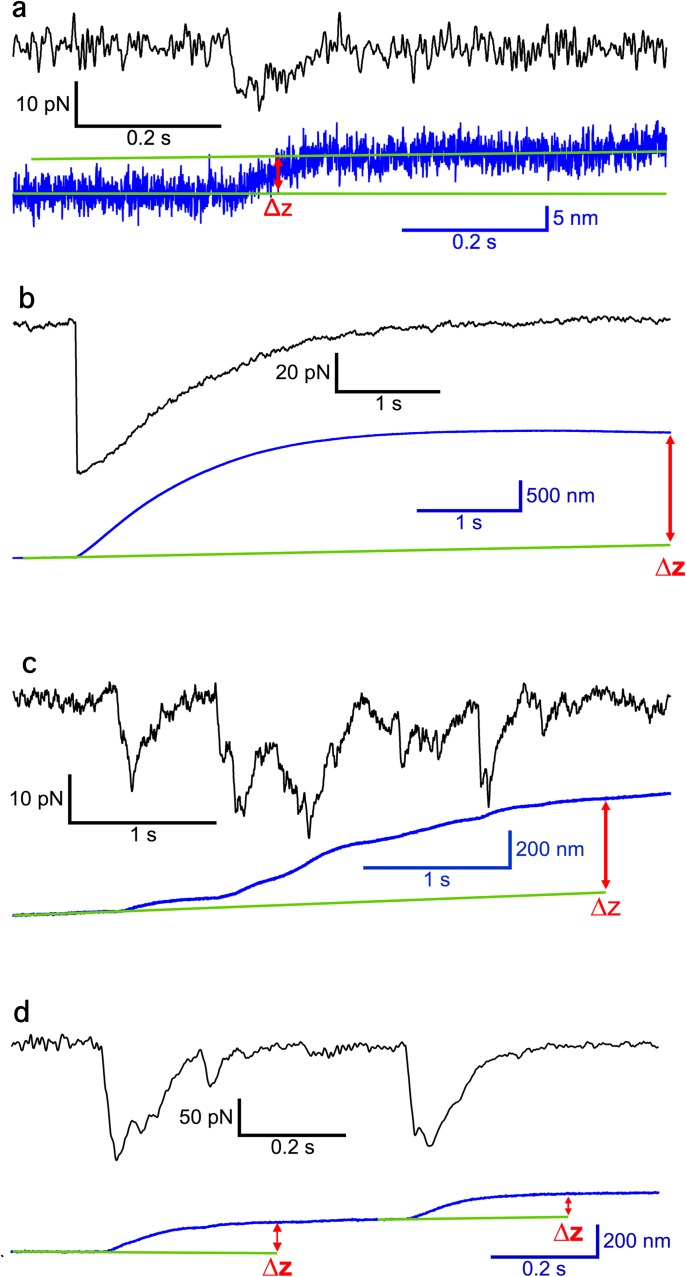
Examples of unitary and complex tether extension events. (a) Unitary small event. The event is characterized by a transient decrease in pull force (black trace) determined from the *V*_*defl*_ change and a simultaneous increase in *z* (blue trace). Note that pull force has opposite sign to *V*_*defl*_. The green line is a projection of the baseline of *z* from before the event to after, and the difference between the projected and observed *z* values is *Δz*, in this case *Δz* = 7 nm. (b) Large unitary event with *Δz* = 1839 nm and single exponential decay. (c) Complex event with many small, overlapping force transients and tether extensions, resulting in total *Δz* = 501 nm. (d) Two complex events with multiple peaks in the force trace, the first with *Δz* = 193 nm, the second with *Δz* = 142 nm. These events may consist of multiple tether extension events that overlap, resulting in the multiple peaks.

Tether extension FC events had a variety of time courses and amplitudes (Fig 6). Most small tether extension events (Fig 6A) and several large events (Fig 6B) showed a force transient with a single exponential decay while some events did not consist of a single peak followed by an exponential decay, but appeared to consist of multiple transients that occurred so rapidly that they overlapped (Fig 6C and 6D). Those events were classified as C (complex) events.

### More force clamp segments show tether extension events in the presence of GTPγS

**Fig 7:**
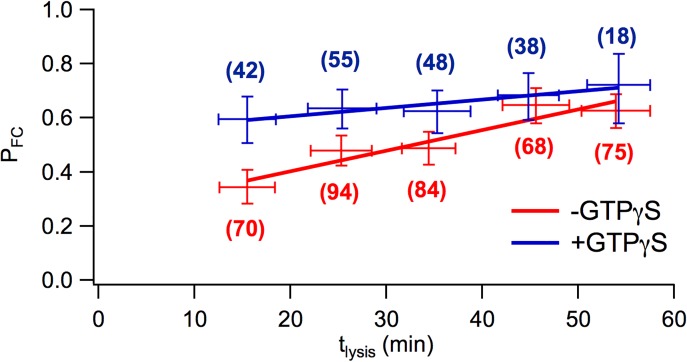
Fraction of segments showing force clamp events at different times after cell lysis. The proportion of segments showing events increases with *t*_*lysis*_ at a rate of ~7.7×10^-3^ min^-1^ in the absence of GTPγS, but starts high and increases at a much lower rate of ~3.1×10^-3^ min^-1^ in the presence of GTPγS. For each segment, time after cell lysis was determined and sorted into 10 min wide bins. For each bin, the mean and SD (horizontal error bars) of t_lysis_ for the segments in that bin were calculated and P_FC_ determined. The vertical error bars are the 68% confidence intervals assuming a binomial distribution. The numbers in parentheses are the number of segments included in each bin.

Vesicle-plasma membrane tethering is regulated by GTP-binding proteins. To interfere with the function of the tethering complex, the occurrence and properties of tether extension events were compared between experiments in the presence (100 μM) and absence of the non-hydrolyzable GTP analog GTPγS. The proportion of segments that contained FC events (*P*_*FC*_) as a function of time after cell lysis (*t*_*lysis*_) (Fig 7) shows that in the absence of GTPγS (-GTPγS), *P*_*FC*_ increased significantly, as determined by unweighted linear regression, up to *t*_*lysis*_ ≈ 60 min, eventually reaching *P*_*FC*_ ≈ 0.6. The higher frequency of tether extension or tether dissociation events in trials performed at longer times after cell lysis indicates that tether stability decreased over time, which is consistent with previous observations of a gradual rundown in the ability of membrane sheets to support exocytosis after cell lysis [16, 17]. In the presence of GTPγS (+GTPγS), *P*_*FC*_ started at ~0.6 followed by a much weaker increase, suggesting that the presence of GTPγS rapidly enabled tether extension events. P_FC_ also increased with increasing pull force up to *F* ≈ 500 pN (S1 Fig).

The proportion of segments showing force clamp tether extension events was slightly higher in the presence of GTPγS for forces *F* < 300 pN, indicating that GTPγS facilitates tether extension events in this force range. For all segments included in the analysis, Pearson’s correlation coefficient between *t*_*lysis*_ and *F* was was found to be 0.014, indicating no significant correlation. Therefore, *t*_*lysis*_ and *F* were treated as independent variables.

### Magnitudes of force clamp tether extension events

For unbiased determination of individual tether extension magnitudes (*Δ*z_tether_), semiautomatic software based on ref. [24] was developed (see S1 Text). The program scans the segments for FC events consisting of a force transient with a simultaneous tether extension and determines the tether extension length Δ*z* for each detected event (Fig 6A). R events were not analyzed because they occurred while the z piezo was in motion, such that tether extension lengths could not be reliably measured.

**Fig 8:**
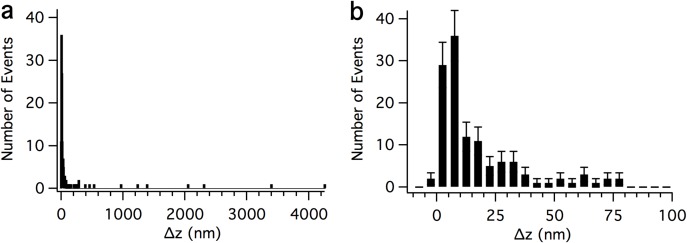
Frequency distribution of tether extension lengths. (**a**) Histogram of all *Δz* values, (**b**) histogram of *Δz* values up to 100 nm, bin width 5 nm. Error bars in (**b**) are the square root of n.

Fig 8 shows histograms of Δ*z* values for all analyzed tether extension events. A wide range of *Δz* values was observed (Fig 8A), but most tether extension lengths were 100 nm or less, with an apparent peak around 5 nm (Fig 8B). The mean Δ*z* was 148 nm for a total of 146 tether extension events. Analysis of events with a Δ*z* < 100 nm returned a mean Δz = 17 nm (n = 123 events). To distinguish between the primary cluster of events with small Δ*z* values and the widely scattered events with larger Δ*z* values, the tether extension events were split into two groups, those with Δ*z* < 50 nm (S or short events), and those with Δ*z* >50 nm (E or extended events). Events with complex time course were accordingly classified C_S_ (complex short) events (extension <50 nm) or C_E_ (complex extended) events (extension >50 nm) (Table 1).

**Table 1:** Classification of force clamp events.

Classification	*Δz*	Appearance in Force Trace
S (short)	<50 nm	Single Peak Transient
E (extended)	>50 nm	Single Peak Transient
C_S_ (complex short)	<50 nm	Complex Transient
C_E_ (complex extended)	>50 nm	Complex Transient

### GTPγS increases frequency of short tether extension events

Individual tether extension events were analyzed in detail for a subset of randomly chosen segments comparing four experimental conditions: +GTPγS and +ΔFluor, +GTPγS and –ΔFluor, -GTPγS and +ΔFluor, and –GTPγS and –ΔFluor. Segments were chosen such that the total force clamp duration included in the chosen segments from each of the four conditions was similar (221 s for +GTPγS and+ΔFluor, 229 s for +GTPγS and –ΔFluor, 238 s for –GTPγS and +ΔFluor, 200 s for –GTPγS and –ΔFluor). Force clamp duration referred to the time elapsed between the pull force initially reaching the force clamp setpoint (after any R events) and the end of the segment or the occurrence of an F event.

**Fig 9:**
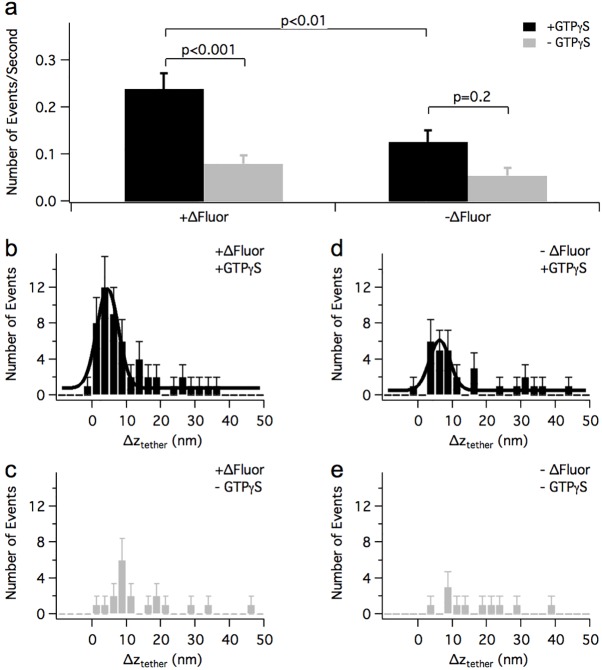
Properties of vesicle-plasma membrane tether extension events. (**a**) Dependence of the frequency of tether extension events <50 nm under force clamp on the presence of GTPγS and ΔFluor. P values were determined using a Poisson test. (**b**-**e**) histograms of Δz_tether_ for conditions as indicated. Histogram bins have width = 2.5 nm and are centered at −8.75 to 48.75 nm. Negative values were allowed to account for measurement noise. Error bars are the square root of the number of events in the bin. The smooth lines in (**b**) and (**d**) are Gaussian fits.

In the presence of GTPγS, the frequency of tether extension events <50 nm was much higher in recordings where a fluorescent vesicle was attached to the AFM tip than in measurements where no fluorescent vesicle was detected (Fig 9A, black bars; +GTPγS +ΔFluor 0.240±0.033 events/s n = 53 events, +GTPγS –ΔFluor 0.127±0.024 events/s n = 29 events). This result indicates that the activation of short tether extension events is specific for vesicle-plasma membrane tethers. There was no difference in the frequency of long (>50 nm) tether extension events among the different experimental conditions. In the absence of GTPγS there was also no significant difference in the frequency of short tether extension events between experiments where a fluorescent vesicle was present or not (Fig 9A, grey bars; –GTPγS +ΔFluor 0.080±0.018 events/s n = 19 events, −ΔFluor –GTPγS 0.055±0.017 events/s n = 11, p = 0.42).

Fig 9B and 9C shows histograms of tether extension lengths (*Δz*_*tether*_) observed for S and C_S_ events that occurred during a force clamp experiment in which a fluorescent vesicle was observed to be pulled by the cantilever tip, while panels d,e show events from experiments with no observable vesicle. When a vesicle was present, the distribution of tether extension lengths (Fig 9B) shows a prominent peak that could be fitted with a Gaussian giving Δz_peak_ = 4.5±0.3 nm and SD(Δz_peak_) = 3.1 nm. In the absence of GTPγS the peak was barely detectable (Fig 9C). GTPγS thus specifically enabled vesicle-plasma membrane tether extension events with extension lengths of ~1-9 nm. When no vesicle was observed at the tip, the distribution obtained in the presence of GTPγS (Fig 9D) showed a small peak with Δz_peak_ = 6.3±0.35 nm and SD(Δz_peak_) = 2.9 nm.

## Discussion

Fusion of secretory vesicles with the plasma membrane is preceded by various steps including tethering, docking, and priming [1, 3]. Long tethers (>5 nm) providing a mechanical link between secretory vesicles and the plasma membrane have been observed by electron microscopy [25], but their mechanical properties are unknown. Here, we developed an approach using AFM force clamp measurements on secretory vesicles of ANF-eGFP expressing PC12 cells associated with plasma membrane sheets that revealed stepwise tether extension events. Simultaneous TIRF imaging of the GFP-labeled vesicles allowed direct detection of vesicle displacement during tether extension induced by mechanical force pulling on the vesicle.

When pulling forces in the range of a few hundred pN were applied, sequential tether extension steps were typically observed. In force clamp experiments in which a fluorescent vesicle was visible at the AFM tip, GTPγS produced an ~3-fold increase in the frequency of short tether extension events (Fig 9A) with a Gaussian distribution of 4.5±3.1 nm (Fig 9B). The frequency of such events was much lower when no fluorescent vesicle was detectable at the AFM tip. These results indicate that the short tether extension events were specifically extensions of the vesicle-plasma membrane tether. In contrast, the frequency of longer tether extension events (>50 nm) was independent of the presence of a fluorescent vesicle or GTPγS, suggesting that they may reflect other interactions of the AFM tip with the cytoplasmic face of the plasma membrane.

A small number of tether extension events with similar length were recorded when no fluorescent vesicle was observed at the AFM tip (Fig 9D). Presumably, these events reflect vesicle-plasma membrane tether extensions in trials where a vesicle was present but was not observed due to low labeling intensity or because it was outside the TIRF excitation field. Fig 3 shows several events that occurred after the vesicle was already pulled out of the evanescent wave. If the vesicle began the run already above the evanescent wave, no change in fluorescence would have been observed, and the experiment would have been labeled as –ΔFluor.

The mechanics of secretory vesicle-plasma membrane tethers revealed by these experiments support the hypothesis that the exocyst complex may be the physical link in the tethered state. The exocyst complex consists of 8 subunits: Sec3, Sec5, Sec6, Sec8, Sec10, Sec15, Exo70, and Exo84 [1]. Exo84 was in fact first identified in PC12 cells [26] and sec6-positive vesicles accumulate at the plasma membrane at sites of cell-cell contact and presumably cell-substrate contact in PC12 cells [27]. The exocyst complex has been shown to function after neurosecretory vesicles have been delivered to exocytic sites, but prior to formation of SNARE complexes [28], which suggests a role of the exocyst during the tethering phase of exocytosis. The exocyst also determines when and where vesicles are tethered [28]. The subunits Exo70 and Sec3 are associated with the plasma membrane, and Sec3 is localized to exocytic sites [29], while the remaining subunits are bound to the vesicle. These findings had led to the hypothesis that assembly of the full complex could form the physical tether [1, 30]. The structures of the four domains of mammalian Exo74 [31] and the Sec6 C-terminal domain [32] have been studied in detail. They consist of helical bundles with α-helices ~25–40 amino acids in length. Assuming an unfolded contour length of 0.365 nm per amino acid [8], and noting that the rise per amino acid of a folded α-helix is 0.15 nm, we expect an extension of 0.215 nm per residue during unfolding or 5.4–8.6 nm to result from the unfolding of a single α-helix. These estimates are in excellent agreement with the range of measured extension lengths observed for the short vesicle-plasma membrane tether extensions in the presence of GTPγS. The position of the peak at 4.5 ± 0.3 nm indicates that some unitary extension events presumably reflect partial unfolding of these helices. It is possible that some larger events represent simultaneous unfolding of multiple domains. Even the existence of multiple short vesicle tethers has been observed associated with vesicles <5 nm from the plasma membrane [25].

Most recently, a model was proposed for the architecture of the exocyst and it was suggested that vesicles may be tethered by multiple exocyst complexes forming a ring surrounding the vesicle-plasma membrane contact zone where fusion occurs [33]. The multiplicity of tether extension events in the pulling experiments described here, may thus involve unfolding events from multiple exocyst complexes tethering a secretory vesicle. Alternatively, it is also possible that the AFM cantilever tip may in some instances bind multiple vesicles and tether extensions in a given pull may involve multiple tethered vesicles.

Fig 3 shows a total extension length of ~3 μm from the beginning of force clamp application until the last extension event in segment 3, which raises the question whether such long extension lengths could be accounted for by unfolding of a single exocyst complex between the vesicle and the plasma membrane. The sequences of the 8 subunits of the rat exocyst complex are available on the NIH Protein database. All subunits are large proteins composed of 653 (exo70) to 975 (sec8) amino acids and the total number of residues of all subunits is 6,216. With an unfolded contour length of 0.365 nm per amino acid, this would give a maximal unfolded length of ~2.3 μm. While this value approaches the total tether extensions obtained in our experiments, it is somewhat smaller, and it seems unlikely that the unfolding of all the exocyst components could produce such a fully extended chain. Furthermore, we observed no difference in the frequency of long (>50 nm) tether extension events among the different experimental conditions, i.e. presence or absence of a fluorescent vesicle or presence or absence of GTPγS. It therefore appears that proteins other than those forming the exocyst must be involved to account for the large extensions. One possibility is actin, as actin is involved in many steps of exocytosis, including an interaction between Myo2, the yeast homologue of Myosin Va, and the exocyst complex in yeast [34]. The role of actin could be investigated in force clamp experiments where cortical actin is disrupted using cytochalasin or latrunculin.

A central finding of our study is a marked increase in the frequency of the ~5 nm tether extension events in the presence of GTPγS, indicating that GTPγS destabilizes tethers and facilitates tether extension. This result further supports the conclusion that these events are specific to vesicle-membrane tethers, due to the multiple roles of GTP-binding proteins in the tethering process, and in particular in the function of the exocyst. The GTP-binding protein sec4p, which is present on secretory vesicles [35], is required for the exocyst complex to fully assemble. Subunit Sec15p binds preferentially to the GTP-bound form of sec4p [36]. When the mammalian ortholog of sec4p, Rab3a [37], is locked in either the GTP-bound or the GDP-bound state in chromaffin cells, the number of vesicles within 100 nm of the plasma membrane decreases, while the number of vesicles found at distances >100 nm from the membrane is not affected [38]. The presence of GTPγS interferes with GTP-GDP cycling, and is therefore expected to interfere with tethering, and potentially to disrupt the fully assembled state of the exocyst, facilitating tether extensions. Additionally, interactions of Exo70 and Sec3 with GTPases from the Rho and cdc42 family are involved in recruitment of the exocyst complex to the plasma membrane [1], and TC10 binds preferentially to the GTP-bound form of TC10 [39].

The small increase in the frequency of 1-9 nm tether extension events produced by GTPγS seen when no fluorescent vesicle was detectable (Fig 9A –ΔFluor, Fig 9D and 9E) could be explained if in some of the -ΔFluor experiments a vesicle may have been pulled on by the cantilever tip, but the vesicle fluorescence was too dim, or the vesicle was disrupted by the AFM tip but its membrane still attached to the AFM tip and tethered to the plasma membrane. Alternatively, such events may have been due to tethers other than the vesicle-plasma membrane tether. GARP, a tethering complex involved in traffic from the endosomes to the trans-Golgi network, has CATCHR morphology, suggesting that tethering mechanisms may be similar for the various membrane trafficking pathways in the cell [40].

Optical trapping experiments in which a half zippered state of the SNARE complex was stabilized showed that unzippering of the N-terminal portion of the SNARE complex results in an extension of 8.3 nm [8]. Since only the N-terminal portion of the SNARE complex is thought to be zippered in the trans state [41], this suggests the possibility that the short tether extension events were due to SNARE complex unzippering. However, the existence of a stable trans state is still being debated [41]. Additionally, unzippering of the SNARE complex cannot account for extension events that occur at distances >10 nm from the plasma membrane, which is the majority of the events described and anlyzed here.

The experiments described here establish a novel approach to measure directly the mechanical properties of vesicle-plasma membrane tethers and their regulation. In future experiments, it could be tested if tetanus toxin, which cleaves synaptobrevin, facilitates tether extensions, which would support the existence of trans-SNARE complexes in the docked state. It has been reported that vesicle tethering still occurs after tetanus toxin treatment, but the duration of tethering events becomes shorter [42]. AFM force clamp experiments on RIM1α knock-out cells could reveal whether the knock-out has a tether destabilizing effect similar to GTPγS. In RIM1α knock-out synapses, the fraction of tethers <5 nm in length decreased [2], possibly representing a disruption of stable tethering that prevents docking. Also, measurement of the extension of purified exocyst subunits, such as Exo70 and Sec6, under AFM force clamp would allow comparison with the stepwise extensions of vesicle-plasma membrane tethers observed in the present experiments and show whether exocyst subunits share the mechanical properties of the vesicle-plasma membrane tether.

## Methods

### Preparation of PC12 cell membrane sheets

Glass-bottomed dishes suitable for TIRF excitation were constructed from 35mm Petri dish lids (#430588, Corning Inc., Corning, NY), into the center of which a 20mm diameter hole was precision cut. A #1.5 30 mm diameter coverslip (64-1499, Warner Instruments, Hamden, CT) was attached to the lid using an RTV615 Silicone Potting Compound kit. To achieve good adhesion of the cell membranes to the surface, a Poly-D-lysine (PDL) coating was applied. 500μL of 0.1% PDL solution (Poly-D-lysine hydrobromide, P7280, Sigma-Aldrich, St. Louis, MO) was pipetted onto the center of a UV-sterilized coverslip, and allowed to incubate for one hour. The dish was then rinsed with sterile H_2_O and air dried for 20 minutes. PDL-coated coverslips were stored at 4°C for up to 7 days.

PC12 cells stably expressing proatrial natriuretic factor linked to eGFP (ANF-eGFP) [43] were obtained from Dr. Ronald Holz, Department of Pharmacology, University of Michigan Medical School. Cells were cultured in F12-K Nutrient mixture with L-Glutamine (21127, Gibco, Life Technologies, Carlsbad, CA) supplemented with 15% horse serum (16050, Gibco), 2.5% Fetal Bovine Serum (10082, Gibco), and 1% Penicillin-Streptomycin-Glutamine 100x (10378, Gibco). To plate the cells in the glass-bottomed dishes, the cells were counted using a Fuchs-Rosenthal chamber and diluted with culture medium to attain an approximate density of 1.6–1.9 × 10^6^ cells/mL. 500μL of this cell suspension was pipetted onto the center of the PDL-coated coverslip. The cultures were incubated at 37°C, 5% CO_2_ for 3 hours. 3mL of growth medium was pipetted into each plated culture. The cells were then allowed to adhere for another 24 hours.

Immediately prior to experiments, the culture medium was replaced by a buffer containing 120mM K-glutamate, 20mM HEPES, 20mM K-acetate, 2mM EGTA, 2mM MgATP, and 0.5mM DTT, pH adjusted to 7.2 with KOH. In some experiments, the buffer was supplemented with 100 μM GTPγS (11 110 349 001, Roche Diagnostics, Indianapolis, IN), obtained as a gift from Dr. Richard Cerione’s lab at Cornell University. The cells were then lysed by a 0.1 s, 20 kHz sonic pulse using a Digital Sonifier 250 (Branson Ultrasonics Corporation, Danbury, CT), with amplitude control at 30%, leaving the cytoplasmic face and associated secretory vesicles exposed [16, 17]. The lysing buffer was removed to wash out debris, and fresh buffer was added.

To label membrane sheets, the styryl dye FM 4-64 (T-3166, Invitrogen, Carlsbad, CA) was added at a final concentration of 0.5 μM after the above steps. The FM 4-64 dye was not used in the experiments in which AFM data were collected.

### TIRF microscopy

For TIRF imaging, an eclipse microscope with a TI-TIRF-E Motorized Illuminator Unit and APO TIRF 100x 1.49NA oil immersion objective (TIRF) (Nikon Corporation, Tokyo, Japan) was used. TIRF images were recorded with a water-cooled iXon EM+ EMCCD camera (Andor Technology, Belfast, Northern Ireland). According to manufacturer specifications, the iXon camera had a pixel size of 16 μm x 16 μm. Accounting for the 100x objective, the pixel size of images recorded by the iXon camera was approximately 160 nm x 160 nm. Andor Solis software recorded the images captured by the iXon camera. The FIRE output of the iXon camera was connected to the “Aux IN” BNC input of the AFM controller and recorded to establish a time correlation between AFM recording and fluorescence images in the analysis. A KP-D250 camera (Hitachi, Tokyo, Japan) was used to visualize the sample and AFM cantilever under bright field illumination, as well as the AFM laser reflection from the cantilever. This was useful for alignment of the AFM laser on the cantilever and for achieving the necessary relative positioning of the cantilever, membrane sheets, and TIRF objective.

The TIRF microscope was equipped with multiple sets of filters from Semrock (Rochester, NY.) For imaging of eGFP excited by a 488 nm Argon-Ion laser (35-LAP-321-120, Melles Griot, Rochester, NY), the following filter set was used: excitation filter FF01-482/18, emission filter FF01-525/45, dichroic Di01-R488. This filter set was also used for bright field illumination. For imaging FM 4-64 excited by a 561 nm diode-pumped solid state laser (CL561-025-O, CrystaLaser, Reno, NV), the following filter set was used: excitation filter FF01-561/14, emission filter FF01-609/54, dichroic Di01-R561. For visualization of the AFM laser reflection from the cantilever, the following filter set was used: excitation filter FF01-390/40, emission filter BLP01-R405, dichroic Di01-R405.

The decay constant of the evanescent wave intensity *d*_*TIRF*_ was determined from force clamp experiments in which a vesicle was attached to the tip during the approach phase (as in Fig 3). For each individual approach, *I* was plotted vs *z* during the approach, and an exponential was fit to data showing a continuous increase in *I* during the approach. The weighted RMS mean and standard deviation of the decay constants obtained from the fits were calculated using the program R, and found to be: *d*_*TIRF*_ = 102±34 nm (mean±sd, n = 5), in good agreement with the theoretical estimate *d*_*TIRF*,*theoretical*_ = 94 nm based on the TIRF illumination angle *θi* = 66.8°.

### AFM force clamp

A 5500 Scanning Probe Microscope (AFM) (Agilent Technologies, Santa Clara, CA) was mounted on the TIRF using an Agilent 5500 ILM Quick Slide stage (S2 Fig). Agilent’s PicoView 1.15 beta software with a custom-written Python script was used for AFM control and data acquisition. The AFM included a piezoelectric servo for fine control of cantilever z position. A position sensor and closed loop feedback corrected for hysteresis.

The membrane sheets, TIRF objective, and AFM cantilever were aligned so that the cantilever tip was positioned over a membrane sheet with vesicles on it and within the TIRF field of view (S1 Text). The membrane sheets were positioned at the focal plane of the objective, and the cantilever tip was ~1 μm above the sheets. The alignment was typically completed within 15 minutes of the time of PC12 cell lysis. The force clamp procedure (Fig 2) was automated with a custom written Python script (for details see S1 Text).

### Data selection for analysis

All force clamp segments were classified by visual inspection of the traces according to the types of events they contained. Since events of different types could occur in a single segment, segment classification represented all types observed. If the cantilever deflection, *V*_*defl*_, did not reach the target setpoint deflection, this indicated that the tip was not in contact with the surface, and the segment was discarded. A segment was labeled as type U (uncertain) if the cantilever tip was not in contact with or pulling on anything during that segment. In such cases, the *V*_*defl*_ signal could still differ from the baseline *V*_*defl*_ because *V*_*defl*_ changed for large *z*_*servo*_ values in the absence of a force bending the cantilever. However, U segments could be clearly identified by the following criteria: no events after the setpoint *V*_*defl*_ was reached, all following segments showed no events of any type, and all *V*_*defl*_ increases during or after the segment in question were accompanied by increases in *z* of approximately 1 μm or more.

The method of calculating pull forces assumed that the cantilever tip was free of all contact with the surface at the times when *V*_*contact*_ and *V*_*end*_ were measured. This may not always have been true, and a large error in *F* could result from a cantilever either beginning or ending a force curve with some deflection due to surface contact. To account for such cases, a small number (Table 2) of segments with *F* events outside of the range of -200–1000 pN were removed, (where a negative *F* value represented a push force). To eliminate the segments with least reliable measurements of *F* while retaining the majority, segments with *σ*_*F*_ > 200 pN were also removed.

**Table 2:** Number of segments remaining in the data set after removal of different segment types.

Segment Type Removed	Number of segments left afterwards
Did not reach target *F* value	1229
*F* > 1000 pN	1146
*F* < -200 pN	1102
*σ*_*F*_ > 200 pN	793
*F* < 0 pN	641
U segments	560

Tether extension or tether dissociation events were not expected to occur at a push force, so all segments in the range of -200–0 pN with an *F* error <200 pN were checked for either R events or *FC* events. In total, 10 out of 152 segments in that range had events. Of those 10 segments, the one with the highest *F* magnitude had *F* = – 31.71 pN. The smallest error in all the segments was 52.15 pN, and the remaining 9 segments had error >160 pN. Thus, there is no significant evidence that any tether extension events occurred at push forces, and segments with *F* < 0 pN were removed from the analysis. Finally, segments of type U were removed, since they did not represent actual pull forces. Table 2 shows the number of total segments included in the data set after each of the above steps.

### Semiautomatic detection and analysis of tether extension events

The algorithm to detect tether extension events detects transients in the applied pulling force traces and subsequently determines the tether extension magnitude. In the originally recorded cantilever deflection voltage signal, which has the reversed sign of the force, the shapes of the force transients associated with tether extension events are similar to those of amperometric spikes indicating single vesicle release events of oxidizable transmitters. The detection of the force transients was thus based on the software developed by Mosharov and Sulzer for amperometric spike analysis [24] (see S1 Text for details). The program determines the times *t*_start_ and *t*_end_ where the force transient starts and ends, respectively.

The tether extension steps coincide with force transients, with the change in *z* starting accordingly at *t*_start_ and *t*_end_ of the force transient. To determine the tether extension step size, a method was implemented to fit a line to user-determined time intervals (default = 200 ms) both before *t*_start_ and *t*_end_, an approach previously developed for the determination of small capacitance step sizes in noisy traces [44]. The estimate of extension step magnitude is taken to be the difference between the values of these fit lines at the time point halfway between *t*_start_ and *t*_end_ (see Fig 6A, for details see S1 Text).

### Statistics

The 68% confidence intervals of the data in Fig 7 and S1 Fig were calculated using the program R’s “binom.confint” command to execute the Pearson-Klopper method. The Pearson correlation coefficient was also calculated using R (http://www.r-project.org). R’s “sample” command was used to choose random segments for analysis. Significance p values of Fig 9A were determined using Poisson test.

## Supporting information

S1 Fig The proportion of segments showing force clamp events at different pull forces. The segments were sorted into bins based on *F*. Data points and error bars indicate for each bin weighted mean and sd of *F*, and mean *P*_*FC*_ with 68% confidence intervals for the binomial distributions. The numbers in parentheses are the number of segments included in each bin. The proportion of segments showing force clamp tether extension events increases with *F* in particular in the absence of GTPγS and tends to be slightly higher in the presence of GTPγS for forces *F* < 300 pN, indicating facilitation of tether extension events by GTPγS.
(PDF)

S2 Fig Diagram of the AFM/TIRF setup. The cantilevers used were Bio-Levers (Olympus, Tokyo, Japan), obtained from Asylum (BL-RC-150VB, Flushing, NY). Asylum reports the Bio-Lever tip radius as ~25±12 nm. For comparison, the radius of PC12 secretory vesicles is ~60 nm [1, 2]. The spring constant was calibrated independently for each cantilever using PicoView’s built-in implementation of the Thermal k method [1, 2] (S1 Text), at a height of 50 μm above the surface of a glass coverslip as used for experiments with buffer but without PDL or cells on it. Values ranged from 0.008 N/m to 0.04 N/m. Asylum reports the spring constant range as 0.009–0.1 N/m, with a typical value of 0.03 N/m. To measure the deflection sensitivity, i.e. the correlation between the physical cantilever deflection and the voltage signal of the quad photodiode, the cantilever was pressed onto the surface of the glass coverslip. Thus, the tip would remain fixed while the servo pressed the cantilever into the surface, causing the cantilever to deflect. A plot of the quad photodiode signal vs servo position yielded the deflection sensitivity. Values could depend on cantilever properties and exact alignment of the AFM laser, and ranged from 33 to 67 nm/V.
(PDF)

S3 Fig Histogram of deflection drifts with servo height (markers) and Gaussian fit. The drift is -0.05±0.043 V/μm. One large drift outlier at -4.78 V/μm is not shown, but it had no significant effect on the fit.
(PDF)

S4 Fig Deflection sensitivity calibration. Panel A: The cantilever is deflected as it is pushed onto a hard surface, changing the angle of the reflected beam of the AFM laser such that it strikes a different spot on the QPD. Panel B: The slope of *V*_*defl*_ vs *z* provides the value of *D*. The cantilever tip is pressed down onto the surface and then retracted, resulting in the appearance of two traces, one recorded as the tip is pushed down, and the other recorded as the tip is retracted.
(PDF)

S5 Fig The *fnum* Trace. The camera FIRE signal was converted to the *fnum* trace, such that the frame number of the TIRF recording was *fnum* × 10^3^.
(PDF)

S6 Fig Automatic detection and analysis of tether extension events. (A) The force transient parameters in terms of *V*_defl_: *V*_max_ is the maximum *V*_defl_ value recorded; *t*(*V*_max)_ is the time at which V_max_ is reached; *t*_1/2_ is the time at which the half maximum is reached. The red lines indicate a linear rise fit and a decaying exponential Fall fit with time constant τ; *t*_*start*_ is the time at which the event begins; *t*_end_ is the time at which the event ends; *V*(*F*_clamp_) is the predetermined stable *V*_defl_ at the applied clamp force baseline value for that experimental segment. (B) The tether extension magnitude from Low Value recorded before *t*_*start*_ to High value recorded for the z-position after *t*_*end*_; Duration indicates the time length of the event; Extension Magnitude of the tether extension event was determined by fitting straight lines to 200 ms segments preceding *t*_start_ and follwing *t*_end_.
(PDF)

S1 Text Supplementary methods. (PDF)
