# Supplementary material for: AFM/TIRF force clamp measurements of neurosecretory vesicle tethers reveal characteristic unfolding steps
Source: PLoS One. 2017 Mar 21;12(3):e0173993. doi: 10.1371/journal.pone.0173993 (PMC5360256; doi:10.1371/journal.pone.0173993)
Supplement: S1 Fig — The segments were sorted into bins based on F. Data points and error bars indicate for each bin weighted mean and sd of F, and mean PFC with 68% confidence intervals for the binomial distributions. The numbers in parentheses are the number of segments included in each bin. The proportion of segments showing force clamp tether extension events increases with F in particular in the absence of GTPγS and tends to be slightly higher in the presence of GTPγS for forces F < 300 pN, indicating facilitation of tether extension events by GTPγS. (PDF) [file pone.0173993.s001.pdf]

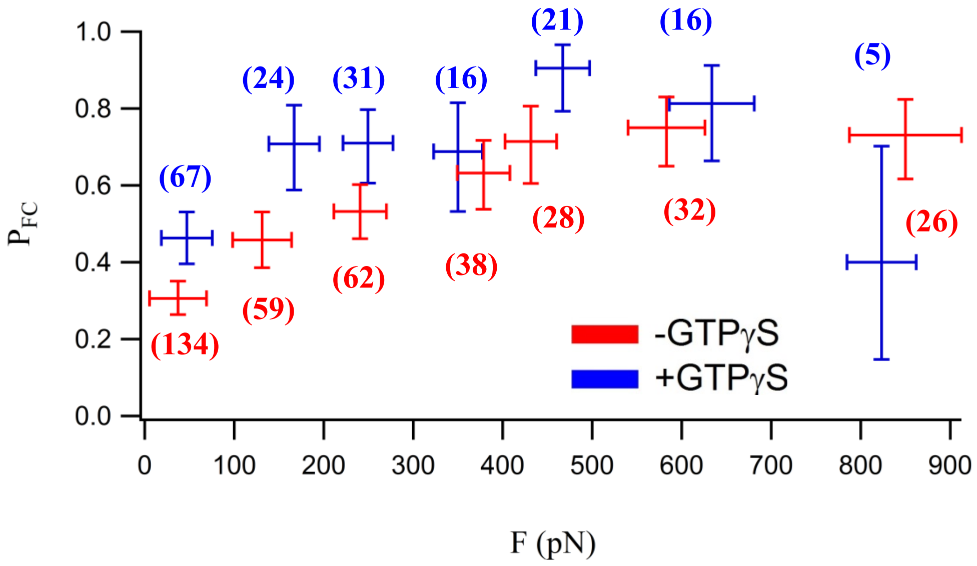

Figure S1. The proportion of segments showing force clamp events at different pull forces. The segments were sorted into bins based on *F*. Data points and error bars indicate for each bin weighted mean and sd of *F*, and mean P_{FC} with 68% confidence intervals for the binomial distributions. The numbers in parentheses are the number of segments included in each bin. The proportion of segments showing force clamp tether extension events increases with *F* in particular in the absence of GTPγS and tends to be slightly higher in the presence of GTPγS for forces $F < 300$ pN, indicating facilitation of tether extension events by GTPγS.
