# Supplementary material for: AFM/TIRF force clamp measurements of neurosecretory vesicle tethers reveal characteristic unfolding steps
Source: PLoS One. 2017 Mar 21;12(3):e0173993. doi: 10.1371/journal.pone.0173993 (PMC5360256; doi:10.1371/journal.pone.0173993)
Supplement: S2 Fig — The cantilevers used were Bio-Levers (Olympus, Tokyo, Japan), obtained from Asylum (BL-RC-150VB, Flushing, NY). Asylum reports the Bio-Lever tip radius as ~25±12 nm. For comparison, the radius of PC12 secretory vesicles is ~60 nm [1, 2]. The spring constant was calibrated independently for each cantilever using PicoView’s built-in implementation of the Thermal k method [1, 2] (S1 Text), at a height of 50 μm above the surface of a glass coverslip as used for experiments with buffer but without PDL or cells on it. Values ranged from 0.008 N/m to 0.04 N/m. Asylum reports the spring constant range as 0.009–0.1 N/m, with a typical value of 0.03 N/m. To measure the deflection sensitivity, i.e. the correlation between the physical cantilever deflection and the voltage signal of the quad photodiode, the cantilever was pressed onto the surface of the glass coverslip. Thus, the tip would remain fixed while the servo pressed the cantilever into the surface, causing the cantilever to deflect. A plot of the quad photodiode signal vs servo position yielded the deflection sensitivity. Values could depend on cantilever properties and exact alignment of the AFM laser, and ranged from 33 to 67 nm/V. (PDF) [file pone.0173993.s002.pdf]

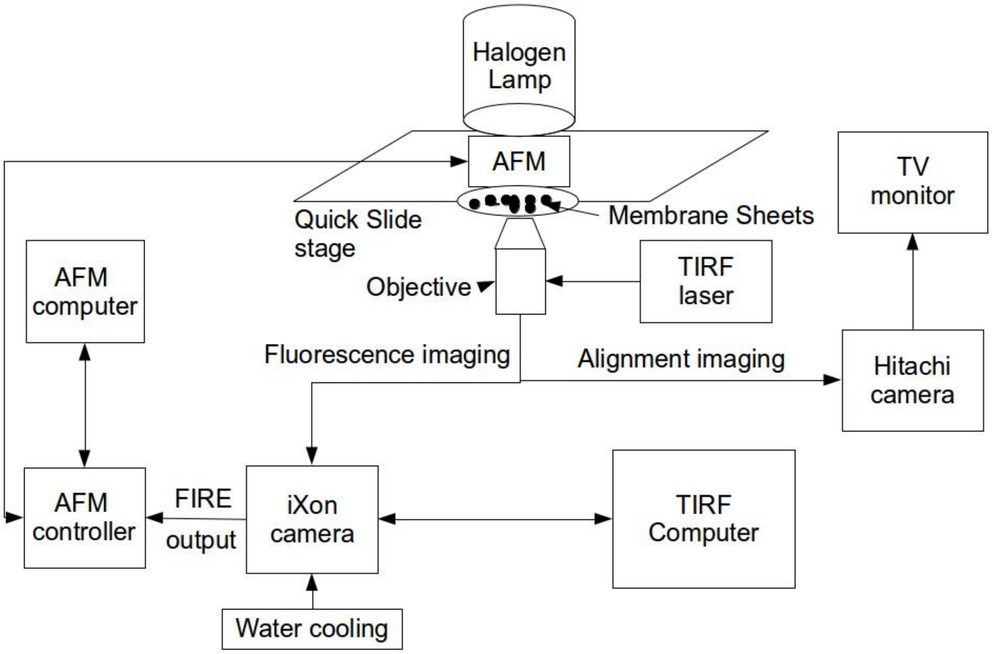


**Figure S2. Diagram of the AFM/TIRF setup.** The cantilevers used were Bio-Levers (Olympus, Tokyo, Japan), obtained from Asylum (BL-RC-150VB, Flushing, NY). Asylum reports the Bio-Lever tip radius as ~25±12 nm. For comparison, the radius of PC12 secretory vesicles is ~60 nm [1, 2]. The spring constant was calibrated independently for each cantilever using PicoView's built-in implementation of the Thermal k method [1, 2] (S7 supplementary methods), at a height of 50 μm above the surface of a glass coverslip as used for experiments with buffer but without PDL or cells on it. Values ranged from 0.008 N/m to 0.04 N/m. Asylum reports the spring constant range as 0.009 – 0.1 N/m, with a typical value of 0.03 N/m. To measure the deflection sensitivity, i.e. the correlation between the physical cantilever deflection and the voltage signal of the quad photodiode, the cantilever was pressed onto the surface of the glass coverslip. Thus, the tip would remain fixed while the servo pressed the cantilever into the surface, causing the cantilever to deflect. A plot of the quad photodiode signal vs servo position yielded the deflection sensitivity. Values could depend on cantilever properties and exact alignment of the AFM laser, and ranged from 33 to 67 nm/V.
