# Supplementary material for: AFM/TIRF force clamp measurements of neurosecretory vesicle tethers reveal characteristic unfolding steps
Source: PLoS One. 2017 Mar 21;12(3):e0173993. doi: 10.1371/journal.pone.0173993 (PMC5360256; doi:10.1371/journal.pone.0173993)
Supplement: S3 Fig — The drift is -0.05±0.043 V/μm. One large drift outlier at -4.78 V/μm is not shown, but it had no significant effect on the fit. (PDF) [file pone.0173993.s003.pdf]

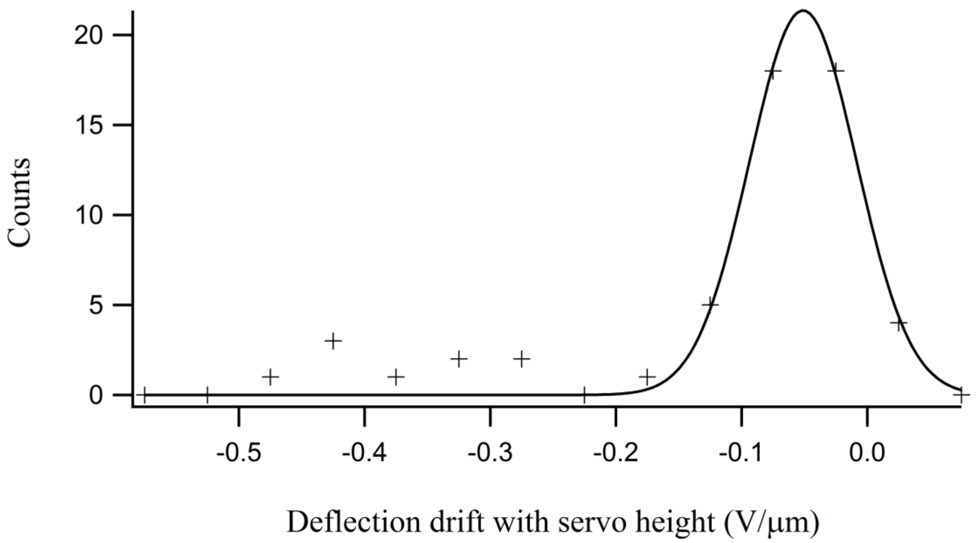

Figure S3. Histogram of deflection drifts with servo height (markers) and Gaussian fit. The drift is -0.05±0.043 V/µm. One large drift outlier at -4.78 V/µm is not shown, but it had no significant effect on the fit.
