# Supplementary material for: AFM/TIRF force clamp measurements of neurosecretory vesicle tethers reveal characteristic unfolding steps
Source: PLoS One. 2017 Mar 21;12(3):e0173993. doi: 10.1371/journal.pone.0173993 (PMC5360256; doi:10.1371/journal.pone.0173993)
Supplement: S4 Fig — Panel A: The cantilever is deflected as it is pushed onto a hard surface, changing the angle of the reflected beam of the AFM laser such that it strikes a different spot on the QPD. Panel B: The slope of Vdefl vs z provides the value of D. The cantilever tip is pressed down onto the surface and then retracted, resulting in the appearance of two traces, one recorded as the tip is pushed down, and the other recorded as the tip is retracted. (PDF) [file pone.0173993.s004.pdf]

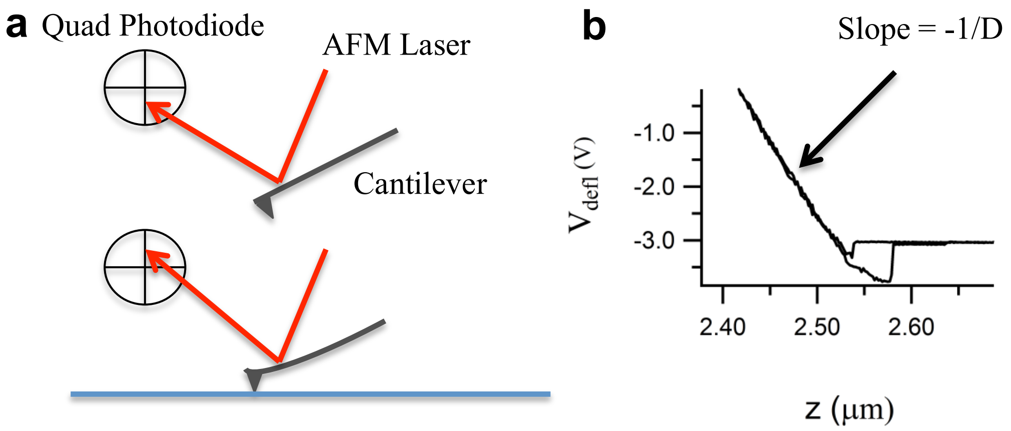

Figure S4. Deflection sensitivity calibration. Panel A: The cantilever is deflected as it is pushed onto a hard surface, changing the angle of the reflected beam of the AFM laser such that it strikes a different spot on the QPD. Panel B: The slope of V_{defl} vs z provides the value of D. The cantilever tip is pressed down onto the surface and then retracted, resulting in the appearance of two traces, one recorded as the tip is pushed down, and the other recorded as the tip is retracted.
