# Supplementary material for: AFM/TIRF force clamp measurements of neurosecretory vesicle tethers reveal characteristic unfolding steps
Source: PLoS One. 2017 Mar 21;12(3):e0173993. doi: 10.1371/journal.pone.0173993 (PMC5360256; doi:10.1371/journal.pone.0173993)
Supplement: S5 Fig — The camera FIRE signal was converted to the fnum trace, such that the frame number of the TIRF recording was fnum × 103. (PDF) [file pone.0173993.s005.pdf]

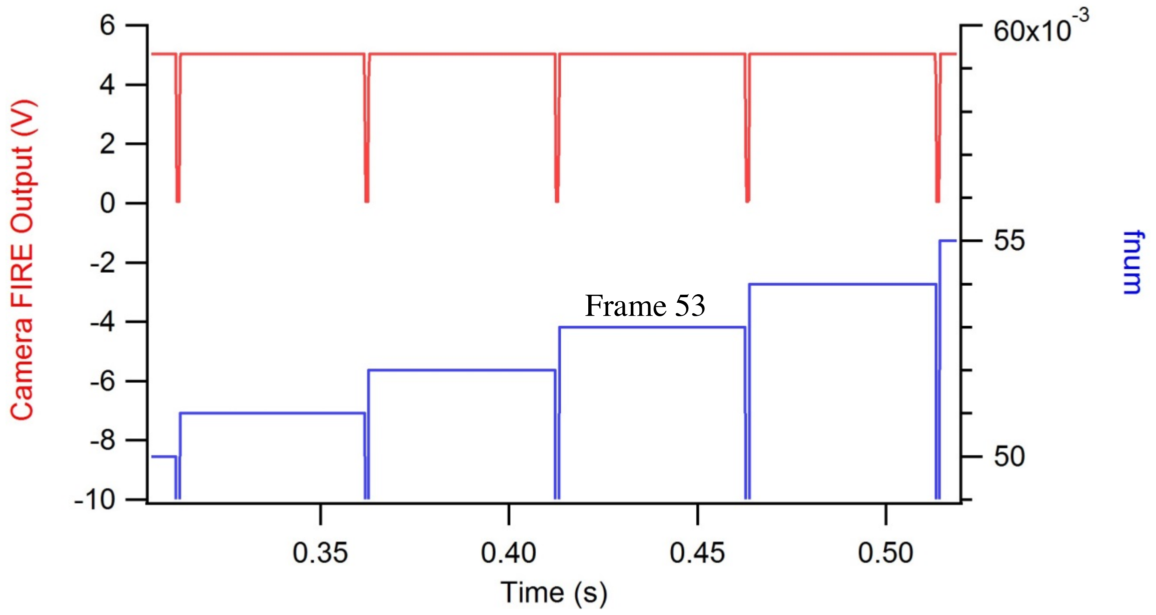

Figure S5. The *fnum* Trace. The camera FIRE signal was converted to the *fnum* trace, such that the frame number of the TIRF recording was *fnum* $\times 10^3$.
