# Supplementary material for: AFM/TIRF force clamp measurements of neurosecretory vesicle tethers reveal characteristic unfolding steps
Source: PLoS One. 2017 Mar 21;12(3):e0173993. doi: 10.1371/journal.pone.0173993 (PMC5360256; doi:10.1371/journal.pone.0173993)
Supplement: S6 Fig — (A) The force transient parameters in terms of Vdefl: Vmax is the maximum Vdefl value recorded; t(Vmax) is the time at which Vmax is reached; t1/2 is the time at which the half maximum is reached. The red lines indicate a linear rise fit and a decaying exponential Fall fit with time constant τ; tstart is the time at which the event begins; tend is the time at which the event ends; V(Fclamp) is the predetermined stable Vdefl at the applied clamp force baseline value for that experimental segment. (B) The tether extension magnitude from Low Value recorded before tstart to High value recorded for the z-position after tend; Duration indicates the time length of the event; Extension Magnitude of the tether extension event was determined by fitting straight lines to 200 ms segments preceding tstart and follwing tend. (PDF) [file pone.0173993.s006.pdf]

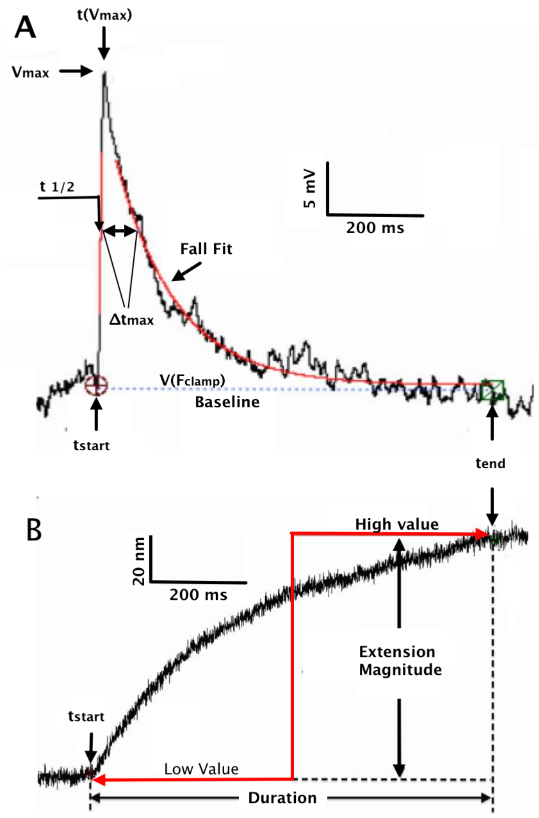

Figure S6. Automatic detection and analysis of tether extension events. (A) The force transient parameters in terms of V_{defl}: $V_{\max}$ is the maximum V_{defl} value recorded; $t(V_{\max})$ is the time at which $V_{\max}$ is reached; $t_{1/2}$ is the time at which the half maximum is reached. The red lines indicate a linear rise fit and a decaying exponential Fall fit with time constant τ; t_{start} is the time at which the event begins; t_{end} is the time at which the event ends; $V(F_{\text{clamp}})$ is the predetermined stable V_{defl} at the applied clamp force baseline value for that experimental segment. (B) The tether extension magnitude from Low Value recorded before t_{start} to High value recorded for the z-position after t_{end}; Duration indicates the time length of the event; Extension Magnitude of the tether extension event was determined by fitting straight lines to 200 ms segments preceding t_{start} and follwing t_{end}.
