# Supplementary material for: AFM/TIRF force clamp measurements of neurosecretory vesicle tethers reveal characteristic unfolding steps
Source: PLoS One. 2017 Mar 21;12(3):e0173993. doi: 10.1371/journal.pone.0173993 (PMC5360256; doi:10.1371/journal.pone.0173993)
Supplement: S1 Text — (PDF) [file pone.0173993.s007.pdf]

S1 Text: Supplementary Methods

AFM force clamp procedure

The force clamp procedure as illustrated in Fig. 2 was automated with a custom written Python script. During the approach and push phases, V_{defl}, servo position, and the iXon camera FIRE signal were all recorded using the Python script at a rate of ~7 Hz. During approach, the cantilever was stepped closer to the surface in 6 nm increments until the measured cantilever deflection, V_{defl}, increased by 0.05V above the initial value, indicating that the cantilever was pressing into the surface. To ensure that the tip was in contact and that the change in V_{defl} was not due to signal drift that was observed to occur both with servo height and with time, the script continued the cantilever approach until two V_{defl} increases of 0.05 V occurred within 50 nm of vertical displacement. These parameters were determined by trial and error to be reliable indicators of surface contact. The approach was performed under TIRF imaging, with the iXon camera acquiring in frame transfer mode with 50 ms exposure times. Approach times were generally <30 s, but varied widely.

Once the cantilever was in contact with the surface, the push phase was initiated, in which the cantilever could bind nonspecifically to the cytoplasmic surface or a vesicle on the surface. The iXon acquisition was terminated, and the cantilever was maintained at constant V_{defl} until a new acquisition was initiated.

In the force clamp phase, the cantilever was retracted to apply 4 different pulling forces of increasing magnitude sequentially, each for 12.5 s. Tether extensions and disruptions could be detected as changes in servo position. If a vesicle moved vertically within the evanescent wave, the extension could also be detected as a decrease in fluorescence intensity. During this phase, V_{defl}, servo position, and the iXon camera FIRE signal were all recorded at a 5 kHz data rate. The

V_{defl} data were later smoothed using Igor Pro's box algorithm at a frequency of 83 points, or about 60 Hz, to remove noise that was observed at that frequency. The iXon recording ended shortly before the last pull force segment ended. The AFM recording thus included the last frame of the exposure, which was later used to identify each individual frame in the FIRE signal (see below section 'Correlation of AFM and TIRF data"). Finally, after the last force clamp segment ended, the servo position was adjusted to 500nm above the position where surface contact was detected during the approach, and a final deflection and time value were recorded.

Conversion of Cantilever Deflection to Force

Typically, conversion of the cantilever deflection V_{defl} to force relies on two calibrations: deflection sensitivity (*D*) and cantilever spring constant (*k*). *D* describes the amount by which the cantilever must be deflected, as measured by vertical displacement of the cantilever tip from rest, to result in a given change in V_{defl}. *k* is the cantilever stiffness, or the force that must be applied to the cantilever to produce a given vertical displacement of the tip. The product of *D* and *k* give the conversion factor, *C*, from V_{defl} to force, such that force = CV_{defl}. In these experiments, there were three sources of error in the measurement of applied pull force that must be taken into account: error in the calibrations *D* and *k*, error due to dependence of baseline V_{defl} on the position, *z*, of the servo that controlled cantilever height, and error in the determination of baseline V_{defl} at the sample surface. Baseline V_{defl} refers to the value of V_{defl} when no force was acting on the cantilever.

To determine the error in *D*, 20 consecutive measurements of *D* were performed with the same cantilever, and the RMS deviation was determined to be 1.88 nm/V. For the force curve

experiments, 5 D values were averaged to determine the value used in the conversion factor, so the final RMS deviation was:

$$\sigma_D = (1.88nm/V)\cdot 5^{-1/2} = 0.841nm/V. \tag{1}$$

The Agilent Thermal k software reported the k value with good reproducibility to the thousandths place in N/m. Therefore, an error of 0.0005 nN/nm was used, or:

$$\sigma_k = 0.0005nN/nm. \tag{2}$$

Thus, the total error in C was:

$$\sigma_C = (k^2\sigma_D^2 + D^2\sigma_k^2)^{1/2}. \tag{3}$$

To examine the dependence of V_{defl} on z, a subset of 60 of the 390 collected force curves was selected using the random number generator in the program R. Of the 60 force curves chosen, 4 were discarded because they were unusable due to problems that occurred during data collection. For the region of a force curve during which the servo was approaching the surface, a linear fit to the V_{defl} vs time trace and the z vs time trace were performed. The ratio of the slopes of the fits gave a value for the V_{defl} drift in $V/\mu m$. A histogram of all the V_{defl} drift values was generated, and a Gaussian fit to the histogram (Fig. S3) had a drift value of -0.05±0.043 V/μm (mean ± sd). To account for the drift effect, each V_{defl} measurement was adjusted by:

$$V_{defl,correction} = z(0.05V). \tag{4}$$

Also, the V_{defl} measurements had an uncertainty of:

$$\sigma_{V_{defl}} = z(0.043V). \tag{5}$$

To confirm that the drift effect was due to servo motion, a histogram of V_{defl} slopes was plotted for the region before the servo was moved (not shown). In this case, the mean drift value was 0.00042 V/μm, suggesting no significant V_{defl} drift.

Baseline V_{defl} at the sample surface was estimated at both the beginning and end of each force curve. During cantilever approach, surface contact was confirmed by an increase of V_{defl} by 0.05 V above the initial value, then a second increase of 0.05 V that occurred with <50 nm further z displacement. The second increase was a test to confirm contact, so the actual point of contact should almost always occur during the first 0.05V increase. Therefore, the V_{defl} at initial contact with the surface was taken to be:

$$V_{surface_i} = V_{contact} - 0.075V \ , \tag{6}$$

where $V_{contact}$ was the value of V_{defl} at which the script reported confirmed contact. Error was then:

$$\sigma_{V_{contact}} = 0.025V \ . \tag{7}$$

A second estimate of baseline V_{defl} was taken after the servo was set to $z_{contact}$ + *500 nm* at the end of each force curve. In this case, the error came from the V_{defl} drift with z, so the baseline V_{defl} at the end was:

$$V_{surface_f} = V_{end} - 0.025V \ . \tag{8}$$

where V_{end} was the V_{defl} value measured at the end of the force curve. The error was:

$$\sigma_{V_{end}} = 0.0215V \ . \tag{9}$$

One more effect had to be accounted for before V_{defl} could be converted to a pull force. V_{defl} was observed to drift negative with time. To account for this, the V_{defl} and time values at surface contact (Eq. 11 and Eq. 13) at the beginning and end of the run were taken as endpoints for a line, and the slope of that line was calculated. For each pull segment, this slope value was multiplied by the time at the segment center and added to the V_{defl} value of that segment. After accounting for the above corrections, the final conversion from V_{defl} to force (F) was:

$$F = -C[V_{defl} + z(0.05V/\mu m) - V_{contact} + 0.075V - \tag{10}$$

$$(V_{end} + 0.25V - V_{contact} + 0.075V)/(t_{end} - t_{contact})](t_{segment} - t_{contact}),$$

where F was the pull force value for the segment, V_{defl} was the deflection value for the segment, $t_{contact}$ was the time at which $V_{contact}$ was measured, t_{end} was the time at which V_{end} was measured, and $t_{segment}$ was the time at the center of the segment. The minus sign was used to give pull forces positive values. When the above errors were included, the variance of each force measurement was:

$$\sigma_F^2 = [\sigma_C \frac{F}{C}]^2 + [\sigma_{V_{defl}} C]^2 + [\sigma_{end} C(t_{segment} - t_{contact})/(t_{end} - t_{contact}) \tag{11}$$

$$+ [\sigma_{contact} C(-1 + (t_{segment} - t_{contact})/(t_{end} - t_{contact}).$$

Alignment of AFM laser and cantilever calibration

The degree of cantilever bending was measured by a laser that was reflected off of the cantilever and onto a quad photodiode (QPD), producing the cantilever deflection signal, V_{defl}. At the beginning of each day of experiments, the AFM laser was aligned on the cantilever such that it was reflected to the approximate center of the QPD. This was done in buffer on the AFM/TIRF assembly using the 405 filter set and Hitachi camera to show the position of the AFM laser on the cantilever. To convert V_{defl} into a pull force, two calibrations were required: deflection sensitivity (D) and cantilever spring constant (k).

D is the amount by which the cantilever must be deflected, as measured by cantilever tip displacement from rest, to result in a given change in V_{defl}. To measure this, the cantilever was pressed onto the surface of a glass coverslip as used for experiments with buffer but without PDL or cells on it. Thus, the tip would remain fixed while z was adjusted, causing the cantilever to

deflect. Fig. S4 shows this procedure and the resulting V_{defl}. A plot of V_{defl} vs z shows a straight line for the region in which the tip is pressed onto the surface, and the slope of that line is $-1/D$. The slope was measured with a procedure built into the PicoView software.

To determine k, the Thermal k method[1,2] built into the PicoView software was used. The Thermal k calibration was performed at a height of 50 μm above the same coverslip with buffer that was used for the deflection sensitivity measurement. To minimize mechanical noise in the power spectrum, the thermal k calibration was performed with the iXon camera water cooling pump turned off.

Alignment of membrane sheets, TIRF objective, and AFM cantilever

Immediately after cell lysis, the coverslip with the membrane sheets was mounted on the AFM, which was slid into place over the TIRF objective using the Quick Slide stage. The halogen lamp was turned on, and the Hitachi camera was used along with a 4x objective and the 488 filter set to observe the cantilever and the lysed region of the sample, where the membrane sheets were located. The lysed region was recognizable by a lack of cells. Micrometers on the AFM stage were then adjusted by hand to position the sample so that the lysed region was located below the cantilever tip, and micrometers on the Quick Slide stage were used to center the AFM tip in the TIRF microscope field of view.

The AFM motor was then used approach the sample surface using the PicoView software. Contact with the sample surface was indicated by V_{defl} exceeding a preset threshold. The AFM tip was centered again in the field of view, using the micrometers on the Quick Slide stage. The 4x objective was switched out for a 40x objective, and the AFM tip was centered again. The 40x objective was then switched out for the TIRF objective. Due to the short working distance of the

TIRF objective, it was no longer possible to view the AFM tip directly. However, by using the 405 filter set, the light from the AFM laser could be seen. The TIRF objective was raised toward the sample until the shadow of the tip was visible within the laser light. When the shadow of the tip was nearly in focus, the objective was close to the sample. The position of the tip was again centered in the field of view, and then the illumination source was changed to the 488 nm laser with the 488 filter set. The eGFP-labeled vesicles were visible in the TIRF illumination. The shadow of the AFM tip could also be seen by allowing a small amount of transmitted light from the microscope's halogen lamp.

The cantilever tip was moved to a new spot away from the original landing spot in case the sample was damaged by the coarse motor approach. To do this, the AFM servo was used to lift the cantilever 1 μm off the surface, and the AFM micrometers were used to move the sample without moving the cantilever. Once a membrane sheet was located, the tip was positioned over it.

Correlation of AFM and TIRF data

For analysis, the V_{defl}, z, and camera FIRE signal traces recorded by PicoView and the Python script were imported into Igor Pro, along with the corresponding time traces. A custom Igor function checked every data point in the FIRE signal. If a point had a value < 3, the camera was considered to not be exposing. Otherwise, the camera was exposing. Using the fact that the last camera frame was known to be frame 1000, a new wave, called *fnum*, was generated that had value equal to the current frame number x 10^{-3} at all points for which the camera was exposing and a lesser value otherwise (Fig. S5). This approach directly matched each camera frame exactly to the corresponding deflection and z sensor data. Using the known cycle time of the

camera (0.05091 s), the frame number of the first full frame after the start of the force segments, and the start time of that frame, the gap between the end of the script data recording and the beginning of the force clamp segments was determined.

During the approach phase, the time resolution of the recording of the FIRE signal was not sufficient to resolve individual camera frames. Therefore, the intensity data was evenly spaced within the timeframe the camera was recording.

Semiautomatic detection and analysis of tether extension events

The detection of the force transients was thus based on the software developed by Mosharov and Sulzer for amperometric spike analysis [3] using the cantilever voltage signal V corresponding to negative force, as illustrated in Fig. S6 using a rather large tether extension event for clarity. First, the program calculates the time derivative of the force trace $V' = \frac{\partial V}{\partial t}$ followed by appropriate smoothing, which facilitates the detection of the rapid rise of the force transient [4]. The time derivatives of all traces analyzed here were smoothed by the Box-Car smoothing method using a box size of 80 data points. This filter was chosen based on the assertion that the primary sources of background fluctuations in the data could be characterized as white noise.

The program searches the derivative trace for the time of a peak, $t(V'_{max})$, above a threshold, m · $SD_{V'}$, where $SD_{V'}$ is the rms noise of V' and m is a user-defined integer parameter. The time of maximal V (minimal force) $t(V_{max})$ is then found as the maximum within the time interval Δt_{max} between $t(V'_{max})$ and the time point having the same or smaller force value on the later descending portion of the transient.

The time points t_{start}, and t_{end} of the force transient (panel a of Fig. S6) are usually taken as the first time points before and after $t(V_{max})$ at which the cantilever deflection voltage (and applied force) returns to the baseline level $V(F_{clamp})$. If $V(F_{clamp})$ is unstable, then this method is

not reliable and the time derivative *V'* is used instead of *V* to determine t_{start}, and t_{end}. In this case t_{start} is set to the time of the first zero of *V'* to the left of $t(V'_{max})$, the time where *V'* has its maximum, and t_{end} is set to the time after $t(V_{max})$ when *V* returns to the level measured at t_{start}. Determinaton of t_{start} and t_{end} is complicated by the broad variability of possible force transient shapes and arrangements. Therefore, a supplemental algorithm was designed to increase the accuracy of t_{start} and t_{end} detection accounting for such complications during automated analysis. If activated, the algorithm will first divide the *V* trace into segments of size Δt_{seg}. The default initial guess for Δt_{seg} is Δt_{max} (see above). Starting at $t(V_{max})$, the program iteratively searches in positive and negative direction until two successive segments are found to have average forces within one SD_V of each other, where SD_V is the rms noise of V. We then set t_{start} and t_{end} as the first time-points at this steady-state force on the corresponding side of $t(V_{max})$,. If subsequent force transients are found to overlap, they are discarded, analyzed by separation, or considered as a single complex tether extension event.

Determination of tether extension magnitudes

Tether extension steps coincide with force transients. Therefore, t_{start} and t_{end} of a tether extension are assigned the time-values obtained from the associated force transient (panel b of Fig. S6). An estimate of the magnitude of a tether extension event can be obtained from the difference between the z-servo position at t_{end} and the z-servo position at t_{start}. While this may be suitable for characterizations of large steps, estimates of the magnitudes of smaller steps become grossly inaccurate due to the noise in the V_{defl} trace.

We therefore developed a more robust approach based on a method originally developed to determine membrane capacitance step sizes associated with vesicle fusion [5] that uses linear extrapolation to find a stable value for the z-position before t_{start} and after t_{end}. The method was implemented to fit a line to user-determined time intervals (default = 200 ms) both before t_{start} and

t_{end}. The estimate of step magnitude is taken to be the difference between the values of these fit lines at the time point halfway between t_{start} and t_{end} (panel b of Fig. S6 and Fig. 6a).

Supplementary References

1. Florin EL, Rief M, Lehmann H, Ludwig M, Dornmair C, et al. (1995) Sensing Specific Molecular-Interactions with the Atomic-Force Microscope. Biosensors & Bioelectronics 10: 895-901.
2. Hutter JL, Bechhoefer J (1993) Calibration of Atomic-Force Microscope Tips. Rev Sci Instrum 64: 1868-1873.
3. Mosharov EV, Sulzer D (2005) Analysis of exocytotic events recorded by amperometry. Nat Methods 2: 651-658.
4. Segura F, Brioso MA, Gomez JF, Machado JD, Borges R (2000) Automatic analysis for amperometrical recordings of exocytosis. J Neurosci Methods 103: 151-156.
5. Dernick G, Gong LW, Tabares L, Alvarez de Toledo G, Lindau M (2005) Patch amperometry: high-resolution measurements of single-vesicle fusion and release. Nat Methods 2: 699-708.
